# A New Clamp

**Published:** 1879-06

**Authors:** 


### A NEW CLAMP.

Dr. E. C. Moore, of Detroit, has devised and made a clamp to hold rubber dam upon the teeth, that for many cases is better than any thing that has preceded it. The edges are more curved than in any other clamps, and pass further on, and even beyond the neck of the teeth in many instances, and thus maintains a very firm hold. It is almost impossible for the patient to throw it off of the tooth by any movement of the mouth or the rubber. I have used them for four months, and would now dislike to be without them. They certainly merit this mention, and a trial in addition.

